# Heart failure with preserved ejection fraction (HFpEF): Diagnosis and management for the general physician

**DOI:** 10.1016/j.clinme.2026.100608

**Published:** 2026-06-22

**Authors:** Rosita Zakeri, Adam A. Nabeebaccus

**Affiliations:** School of Cardiovascular and Metabolic Medicine & Sciences, Faculty of Life Sciences and Medicine, King’s College London, UK

**Keywords:** Heart failure with preserved ejection fraction, HFpEF, Diagnosis, Management, Multimorbidity, Echocardiography, Natriuretic peptides, Cardiometabolic disease

## Abstract

Heart failure (HF) with preserved ejection fraction (HFpEF) is becoming the dominant HF phenotype in clinical practice, reflecting population ageing and increasing cardiometabolic disease. Contemporary HFpEF is recognised as a complex, systemic syndrome with heterogeneous pathophysiology and substantial morbidity. It remains underdiagnosed, with many patients detected only during hospitalisation. Diagnosis requires a high index of suspicion and a structured approach integrating clinical assessment, natriuretic peptides and echocardiography to demonstrate elevated left ventricular filling pressures and exclude alternative diagnoses. Recent randomised trials have demonstrated that sodium-glucose cotransporter-2 (SGLT2) inhibitors and non-steroidal mineralocorticoid receptor antagonists reduce HF hospitalisations and improve quality of life. Additional strategies, including obesity-targeted therapies, support phenotype-directed management, alongside core strategies of congestion relief, comorbidity optimisation, and multidisciplinary care. Early recognition and timely initiation of evidence-based therapy are essential to improve outcomes for this population. This review provides a practical, evidence-based framework for the contemporary diagnosis and management of HFpEF.

## Highlights

• HFpEF is a common, under-recognised and heterogeneous syndrome that should be considered in patients with unexplained dyspnoea and cardiometabolic risk factors.
• Diagnosis requires a structured approach combining clinical assessment, measurement of natriuretic peptides and echocardiography to confirm elevated left ventricular filling pressures and exclude alternative causes of symptoms.
• SGLT2 inhibitors are the principal evidence-based therapy, reducing HF hospitalisations and improving symptoms; non-steroidal mineralocorticoid receptor antagonists also reduce HF events.
• Cardiometabolic therapies, including glucagon-like peptide-1 receptor (GLP-1) agonists, improve symptoms and quality of life in patients with HFpEF and obesity.
• Management should also prioritise decongestion, comorbidity optimisation and multidisciplinary care. Early recognition and treatment can meaningfully improve symptoms and reduce adverse outcomes in this population.

## Introduction

Heart failure (HF) is a clinical syndrome defined by characteristic symptoms and signs resulting from cardiac structural and/or functional abnormalities.^1^, ^2^ It has long been recognised that a substantial proportion of patients exhibiting HF have preserved left ventricular (LV) ejection fraction (EF, commonly defined as a LVEF ≥50%).^3^ The previous nomenclature of ʻdiastolic HF’ lacked specificity and has been superseded by HF with preserved ejection fraction (HFpEF), reflecting its recognition as a complex, multisystem syndrome rather than isolated diastolic dysfunction.

HFpEF is now the predominant HF phenotype in the population, frequently encountered in patients with multimorbidity and across non-cardiology settings, due to overlapping symptom profiles. Outcomes remain poor, with morbidity and mortality comparable to HF with reduced ejection fraction (HFrEF), alongside high rates of healthcare utilisation and reduced quality of life. Recent randomised controlled trials (RCTs) have demonstrated that HFpEF is a treatable condition, therefore timely recognition and accurate diagnosis are essential to enable evidence-based therapy and improve clinical outcomes.

This review summarises contemporary understanding of HFpEF, including evidence from RCTs and guideline recommendations, and provides a practical framework for the diagnosis and management of HFpEF.

## Epidemiology

HFpEF affects between 1.1% and 5.5% of the general population and accounts for around half of all HF cases, although the true proportion varies depending on the population studied and diagnostic criteria used.^4^ Its prevalence is rising faster than that of HFrEF, reflecting its strong association with population ageing and the rising prevalence of its major risk factors: obesity and cardiometabolic disease. HFpEF is also under-recognised. Screening studies report a substantial prevalence of HFpEF among individuals with unexplained dyspnoea or previously unrecognised HF in the community.^5^

The prognosis is not benign. Although earlier studies suggested more favourable survival than HFrEF, contemporary studies indicate broadly comparable outcomes, with a greater proportion of non-cardiovascular causes of death in older HFpEF populations.^6^ Patients living with HFpEF experience impaired quality of life, reduced functional capacity and recurrent hospitalisations, contributing to high healthcare utilisation. Importantly, many patients report symptoms before their first hospitalisation.^7^ Therefore, earlier diagnosis represents an important opportunity to improve outcomes.

## Pathophysiology

HFpEF is pathophysiologically distinct from HFrEF. Its defining haemodynamic feature is elevated LV filling pressures at rest or during exertion, arising from a complex interplay of cardiac and extra-cardiac mechanisms.

### Dominant pathophysiological mechanisms

The traditional paradigm of isolated LV afterload excess has evolved; HFpEF is now understood to reflect impaired reserve across multiple cardiovascular domains. Abnormal ventricular–vascular coupling, concentric remodelling (ie increased LV wall thickness relative to LV cavity size) with or without overt hypertrophy, myocardial fibrosis, coronary microvascular dysfunction, impaired lusitropy, chronotropic incompetence and reduced systolic reserve collectively limit the ability to augment cardiac output during stress, leading to exertional intolerance and congestion.^8^

Paulus and Tschöpe further reframed HFpEF as a systemic cardiometabolic inflammatory syndrome, in which comorbidities promote endothelial dysfunction, reduced nitric oxide-cGMP-PKG signalling and myocardial stiffening.^9^ Notably, myocardial dysfunction in HFpEF is not confined to the LV. Abnormalities of the left atrium (LA), pulmonary vasculature and right ventricle are common and prognostically relevant.^10^, ^11^ Left atrial dysfunction contributes to symptoms and is closely linked to atrial fibrillation (AF).^10^ Pulmonary hypertension is initially passive, but may progress to intrinsic pulmonary vascular disease and right ventricular impairment.^11^

### Phenotypic heterogeneity

**Fig. 1:**
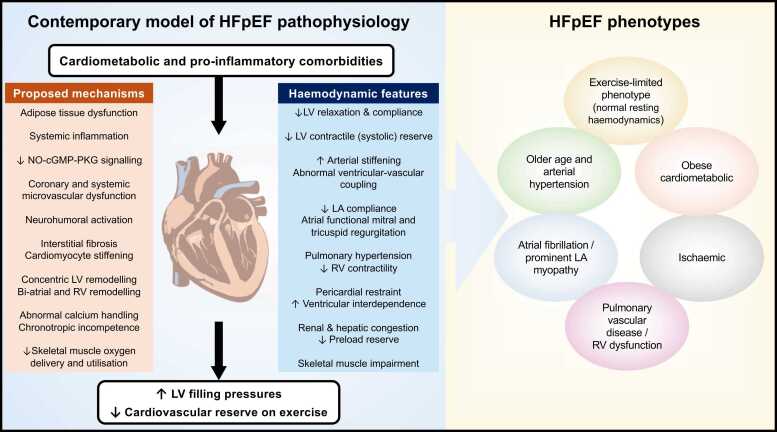
Contemporary model of HFpEF pathophysiology and phenotypes. HFpEF is recognised as a multisystem syndrome driven by pro-inflammatory cardiometabolic comorbidities, leading to microvascular dysfunction, myocardial stiffening and adverse remodelling. The end result is elevated left ventricular filling pressures and reduced cardiovascular reserve. Heterogeneous HFpEF phenotypes have been identified; those displayed represent commonly described, overlapping but non-exhaustive groups with varying dominant underlying mechanisms.

Contemporary HFpEF is recognised as a heterogeneous syndrome with overlapping phenotypes, unified by elevated LV filling pressures arising through distinct dominant mechanisms (Fig. 1). In addition to the ʻclassic’ hypertensive phenotype, often seen in older women, a cardiometabolic HFpEF phenotype, characterised by obesity, diabetes and multimorbidity, has become increasingly common and may present at a younger age.^12^ More recently, an adiposity-based paradigm of HFpEF has emerged, in which dysfunctional adipose tissue and adipokine signalling are proposed to contribute to the pathophysiology of obesity-related HFpEF.^13^ Although data-driven phenotyping reveals marked biological and clinical heterogeneity of HFpEF, such classifications have not yet translated into routine clinical decision-making.^12^

A useful clinical distinction is between patients with overt congestion and those with exertional symptoms without clear fluid overload. The latter often represent earlier or less overt disease and can be challenging to recognise in routine practice.^14^

## Diagnosis

**Fig. 2:**
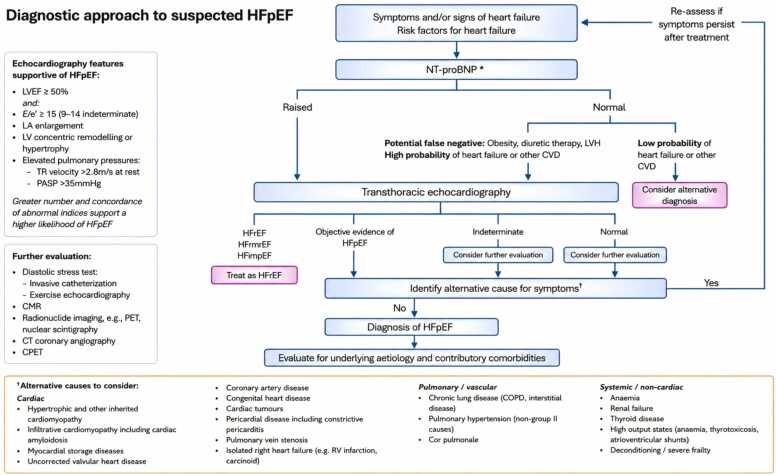
Diagnostic approach to suspected HFpEF. The algorithm reflects contemporary guideline recommendations where diagnosis requires the presence of: i) symptoms and/or signs of HF, ii) preserved LV ejection fraction (≥50%), and iii) objective evidence of elevated left ventricular filling pressures primarily demonstrated using natriuretic testing and transthoracic echocardiography, in line with European Society of Cardiology (ESC) criteria. Where diagnostic uncertainty persists, further evaluation is recommended, alongside consideration of alternative causes of symptoms. *Abbreviations:* CMR, cardiac magnetic resonance imaging; CPET, cardiopulmonary exercise test; CT, computed tomography; CVD, cardiovascular disease; ECG, electrocardiogram; HFimpEF, heart failure with improved ejection fraction; HFmrEF, heart failure with mildly reduced ejection fraction (LVEF 40–49%); HFrEF, heart failure with reduced ejection fraction (LVEF <40%); LA, left atrial; LV, left ventricular; LVEF, left ventricular ejection fraction; LVH, left ventricular hypertrophy; PASP, pulmonary artery systolic pressure; PET, positron emission tomography; TR, tricuspid regurgitation. *NT-proBNP should be interpreted alongside standard investigations including 12-lead ECG, routine blood tests, ± chest X-ray and ± ambulatory ECG.

HFpEF is a diagnosis of probability based on concordant clinical, biomarker and imaging findings. No single non-invasive test is diagnostic; therefore, a structured approach is required (Fig. 2).

### When to suspect HF and refer

HFpEF typically presents with exertional dyspnoea, reduced exercise tolerance and fatigue, most commonly in older patients with cardiometabolic multimorbidity (eg hypertension, obesity, diabetes, AF, chronic kidney disease (CKD)). Orthopnoea is relatively specific but insensitive. Signs of congestion may be absent in early disease or in patients already receiving diuretics. A diagnosis of HFpEF should be considered in patients with unexplained exertional dyspnoea, particularly those with cardiometabolic risk factors.

In primary care, UK NICE guidelines recommend NT-proBNP testing, with referral for echocardiography and specialist review if NT-proBNP ≥400 ng/L (urgent pathways for NT-proBNP >2,000 ng/L or previous myocardial infarction).^15^ These referral thresholds are higher than the natriuretic peptide cut-offs used in major international diagnostic guidelines, including those from the ESC and ACC/AHA/HFSA, which use NT-proBNP ≥125 ng/L to support the diagnosis of chronic HF.^1^, ^16^ Low natriuretic peptide levels have a high negative predictive value for excluding HF; however, values may be lower in obesity and higher in AF, CKD and advancing age, and may be elevated in the absence of HF. Therefore, interpretation of natriuretic peptide levels requires clinical context. If clinical suspicion remains high despite a low NT-proBNP level, echocardiography should be performed.

### Echocardiography and initial assessment

Echocardiography is essential but is not diagnostic of HFpEF in isolation. Typical findings include a non-dilated LV with preserved LV ejection fraction, concentric remodelling and LA enlargement. However, most echocardiographic features lack specificity and may be present in individuals without HF, therefore findings must be interpreted in the clinical context. Echocardiography also plays a key role in excluding alternative causes of dyspnoea, including HFrEF, valvular disease and pericardial pathology.

### Confirming the diagnosis

The central principle is to demonstrate a cardiac explanation for symptoms, supported by objective evidence of elevated LV filling pressures. The latter includes elevated natriuretic peptides and cardiac structural or functional abnormalities on echocardiography. Diagnostic confidence increases with the number and concordance of abnormal echocardiographic parameters. Recent consensus statements support adjusted thresholds in patients in AF and use of additional echocardiographic parameters such as LA strain and measures of subclinical LV systolic dysfunction such as global longitudinal strain (GLS).^17^

### When the diagnosis remains uncertain

In patients with exertional symptoms and inconclusive resting investigations, further evaluation is required. This may include diastolic stress echocardiography or invasive haemodynamic assessment, which remains the reference standard. Exercise echocardiography is considered abnormal if the average E/e′ ratio at peak stress increases to ≥15, with or without a peak tricuspid regurgitation (TR) velocity >3.4 m/s. On invasive testing, elevated LV filling pressures at rest (eg pulmonary capillary wedge pressure (PCWP) ≥15 mmHg or LV end diastolic pressure (LVEDP) ≥16 mmHg) or during exercise (eg peak exercise PCWP ≥25 mmHg) are consistent with HFpEF. Invasive testing is less commonly performed but may be useful in selected cases when diagnostic uncertainty persists after non-invasive assessment or where there is suspicion of concomitant obstructive coronary artery disease (CAD) or pulmonary hypertension, and haemodynamic characterisation would influence management. Additional investigations may be required to identify underlying or contributory conditions, such as cardiac MRI, ambulatory rhythm and blood pressure monitoring.

### Role of diagnostic scores

Diagnostic scores such as H_2_FPEF and HFA-PEFF can provide estimates of HFpEF probability by integrating clinical, biomarker and echocardiographic data (Table 1). However, their performance varies across populations, and these scores are currently best considered adjuncts to clinical assessment rather than definitive diagnostic tools.^18^, ^19^ They do not replace clinical judgement. Newer scores (eg HFpEF-ABA, BREATH_2_) may aid early recognition in non-specialist settings but are not yet widely validated or guideline-endorsed.

**Table 1:** Diagnostic scoring systems to support recognition of HFpEF.

Score	Components	What it assesses	Interpretation	Strengths	Limitations/clinical caveats	Intended use
**H₂FPEF score**^20^	**H₂FPEF**: • **H**eavy (BMI >30 kg/m^2^) • **H**ypertension (≥2 drugs) • **A**trial fibrillation • **P**ulmonary hypertension (PASP >35 mmHg) • **E**lder (age >60 y) • **F**illing pressures (E/e′ >9)	Clinical + echocardiographic likelihood of HFpEF	Score 0–9: • 0–1: low probability • 2–5: intermediate • 6–9: high probability	• Simple and quick • Uses routine data • Good initial screening tool	• Less accurate in early/exertional HFpEF • May overestimate in obesity/AF • No natriuretic peptides included	First-line in clinic/echo setting to estimate probability
**HFA-PEFF algorithm**^21^**(ESC/HFA)**	Domains: • Functional (E/e′, TR velocity) • Structural (LA size, LV mass) • Biomarkers (NT-proBNP)	Multidomain diagnostic framework	Score 0-6: • 0–1: unlikely • 2–4: indeterminate → further testing • 5–6: diagnostic	• Comprehensive • Guideline-aligned • Incorporates biomarkers	• More complex • Many patients indeterminate • Requires high-quality echo	Preferred in secondary/specialist care
**HFpEF-ABA score**^22^	Variables include: • Age • Body mass index • Atrial fibrillation	Clinical risk-based probability model	Higher score → higher likelihood of HFpEF	• Very simple • Purely clinical (no echo required) • Useful in early triage	• Limited validation compared with H₂FPEF/HFA-PEFF • Does not assess cardiac structure/function	Primary care/screening settings
**BREATH₂ score**^23^	Includes: • BNP/NT-proBNP • Renal function • Echocardiographic parameters • Age and comorbidities	Integrates biomarkers + imaging to assess likelihood of HFpEF	Probability-based (low/intermediate/high likelihood)	• Incorporates natriuretic peptides • Reflects multimorbidity burden • May improve discrimination in dyspnoea pathways	• Less widely used • Requires biomarker + echo data • Limited real-world uptake	Potential role in diagnostic pathways for unexplained dyspnoea
**General clinical assessment**^1^	Symptoms, signs, NT-proBNP, echocardiography	Establishes suspicion and need for referral	Not formally scored	• Reflects real-world practice • Essential first step	• Non-specific • HFpEF often under-recognised	Primary care and acute settings

BMI, body mass index; LA, left atrial; LV, left ventricular; PASP, pulmonary artery systolic pressure; TR, tricuspid regurgitation.

### Special considerations and diagnostic pitfalls

A critical step is the exclusion of HFpEF mimics, ie conditions that present with HF and preserved ejection fraction but have distinct pathophysiology and management. Examples include cardiac amyloidosis, hypertrophic cardiomyopathy, constrictive pericarditis and significant valvular disease.

Normal natriuretic peptide levels do not exclude HFpEF, particularly in obesity, where circulating natriuretic peptide concentrations are often suppressed.^24^, ^21^ Natriuretic peptide release is primarily driven by increased LV end-diastolic wall stress, which is inversely proportional to wall thickness. Therefore, natriuretic peptide levels may also be low in the setting of LV hypertrophy or in patients who are well diuresed. Clinical judgement remains paramount when suspicion is high.

Patients with HF and LV ejection fraction between 40% and 49% (HF with mildly reduced ejection fraction, HFmrEF) or LV ejection fraction ≥50% with a previously documented LV ejection fraction <50% (HF with improved ejection fraction, HFimpEF) should be considered separately, as their biology aligns more closely with HFrEF. These patients should receive guideline-recommended therapy for HFrEF.

Pulmonary hypertension is common in HFpEF but should still prompt evaluation for alternative or additional causes, including lung disease and chronic thromboembolic disease. Constrictive pericarditis should be considered when features of right heart failure are disproportionate to left-sided disease, particularly if there is a history of cardiac surgery or thoracic radiotherapy.^25^

In practice, the greatest challenge is often distinguishing HFpEF from non-cardiac dyspnoea or deconditioning in patients with multimorbidity. Subtle symptoms may be misattributed to ageing, while sedentary behaviour may mask functional limitation. Therefore, HFpEF should be actively considered in patients with unexplained exertional symptoms or limitation, particularly where risk factors for HFpEF are present.

## Chronic management of HFpEF

**Fig. 3:**
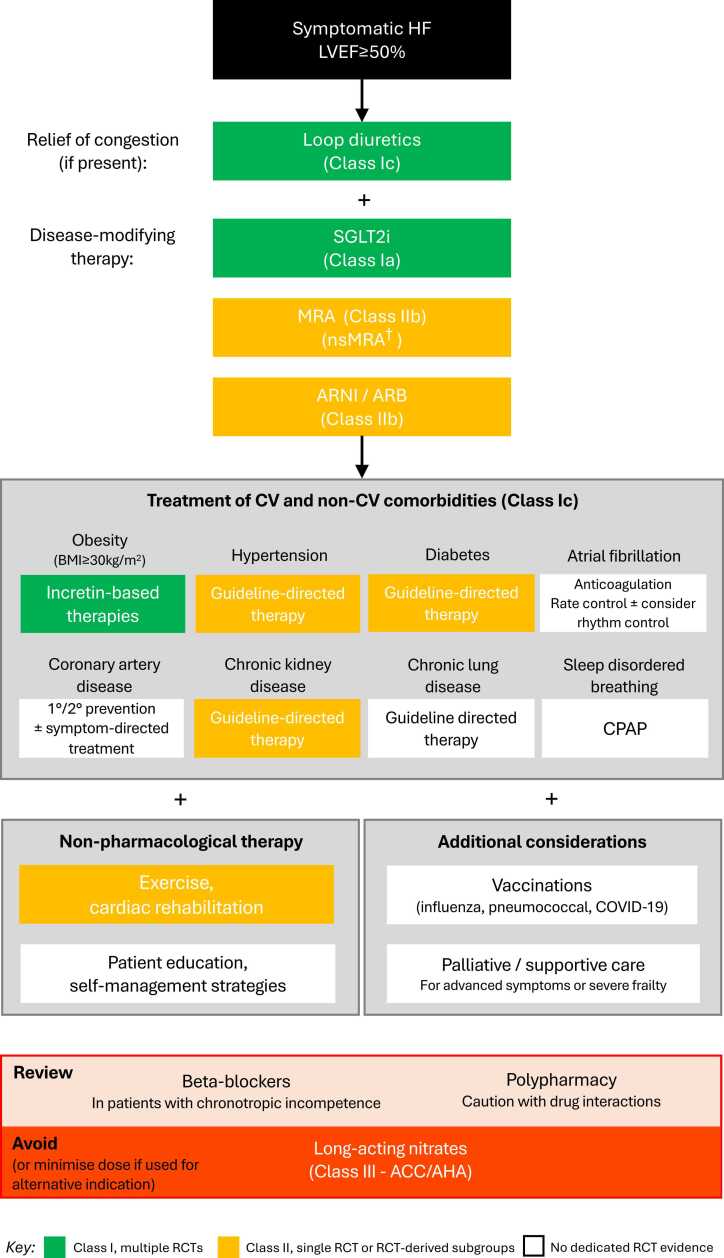
Overview of chronic HFpEF management. Pragmatic approach to chronic HFpEF management, incorporating decongestion, evidence-based therapies, comorbidity optimisation, and non-pharmacological care, in line with contemporary guidelines and tailored to the individual clinical phenotype. Recommendation classes and levels of evidence are shown where applicable. Recommendation classes reflect the strength of guideline recommendations (Class I = recommended; Class IIa = should be considered; Class IIb = may be considered; Class III = not recommended). Levels of evidence (A–C) reflect the quality and quantity of supporting evidence. *Abbreviations:* ACC/AHA, American College of Cardiology/American Heart Association; CPAP, continuous positive airway pressure; HF, heart failure. ^†^Evidence for non-steroidal MRAs is emerging; HFpEF guideline recommendations are awaited.

Current HFpEF therapies primarily reduce the risk of hospitalisation rather than mortality and, in contrast to HFrEF, disease-modifying therapies remain limited. Management therefore centres on relief of congestion, optimisation of comorbidities, and improvement in symptom burden and functional status (Fig. 3).

## For all patients

### Relief of congestion

Loop diuretics are the cornerstone of therapy for clinical or subclinical fluid overload (ESC Class I, level C).^1^ Despite limited trial data, they are effective for symptom relief and prevention of hospitalisation. The lowest dose required to achieve and maintain euvolaemia should be used, with regular monitoring of renal function and electrolytes.^15^ In patients with persistent congestion despite adequate loop diuretic therapy, sequential nephron blockade, with the addition of a thiazide or thiazide-like diuretic (eg bendroflumethiazide or metolazone) may be considered to augment natriuresis. Monitoring is required due to the risk of hypokalaemia, hyponatraemia and worsening renal function. Mineralocorticoid receptor antagonists (MRA) may augment diuretic response in selected patients. SGLT2 inhibitors exert modest osmotic diuresis but are not primarily used for decongestion.

In the CHAMPION trial, remote haemodynamic monitoring reduced HF hospitalisations compared with standard care, largely through earlier and more responsive adjustment of diuretic therapy, highlighting the central role of congestion management in HFpEF.^26^

### SGLT2 inhibitors

SGLT2 inhibitors are a cornerstone pharmacological therapy for HFpEF (ESC 2023 Class I, Level A^1^; NICE 2025^15^), independent of diabetes status. The EMPEROR-Preserved^27^ and DELIVER^28^ trials demonstrated consistent reductions in the primary composite endpoint of HF hospitalisation or cardiovascular death in patients with HF and LVEF >40%, with the benefit driven predominantly by reductions in HF hospitalisation rather than cardiovascular mortality. Absolute risk reductions were modest, nevertheless, the time to therapeutic benefit appears early, within weeks, emphasising the importance of early initiation. Improvements in quality of life are modest at a population level but clinically meaningful in a significant proportion of patients. Additional benefits include attenuation of renal function decline.

SGLT2 inhibitors are straightforward to initiate, require minimal monitoring and can be started early, including after hospitalisation. Caution is required in patients with diabetes treated with insulin due to the risk of ketoacidosis, and specialist input is appropriate in such cases.

### Mineralocorticoid receptor antagonists

Evidence for steroidal MRAs in HFpEF remains equivocal. The neutral overall result of the TOPCAT trial evaluating spironolactone^29^ was attributed to regional heterogeneity, with benefits reported for the subgroup of patients enrolled in the Americas, who had higher event rates.^30^ Consequently, several international guidelines (US, Canadian, Japanese) provide a Class IIb recommendation (‘may be considered’) for selected patients. The 2025 NICE guidance similarly supports the use of MRAs in HFpEF, without specifying agent or phenotype.^15^

The non-steroidal MRA finerenone has demonstrated cardiovascular and renal benefit in patients with CKD and type 2 diabetes. More recently, FINEARTS-HF showed that finerenone reduced worsening HF events and cardiovascular death in patients with HFmrEF and HFpEF, representing the first positive outcomes trial of an MRA in this population.^31^ Finerenone is recommended by NICE for selected patients with CKD,^32^ and its role in HFpEF is under evaluation.^33^ In current practice, it may be particularly relevant in patients with coexistent HFpEF, diabetes and CKD, where indications overlap.^32^ Ongoing studies are evaluating finerenone in acute HF and non-diabetic CKD. In all cases, MRA therapy requires monitoring of renal function and potassium, particularly in patients with CKD.

### Angiotensin receptor/neprilysin inhibitors

The role of angiotensin receptor/neprilysin inhibitors (ARNI) and other renin-angiotensin aldosterone system (RAAS) inhibitors in HFpEF is restricted to selected subgroups. In the PARAGON-HF trial, sacubitril/valsartan did not significantly reduce the primary composite outcome of HF hospitalisation or CV death in patients with HF and LVEF ≥45%,^34^ although prespecified analyses suggested greater benefit in women and in those with LVEF at the lower end of the preserved spectrum. The PARALLAX trial showed no improvement in functional capacity (6-min walk distance).^35^

Current guidelines suggest that ARNI may be considered in selected patients (Class IIb), particularly those with LVEF at the lower end of the preserved range or coexistent hypertension.^16^ Treatment should be individualised, with monitoring of blood pressure (risk of hypotension) and renal function.

### Therapies to avoid or review

Long-acting nitrates should not be prescribed specifically for HFpEF, as the NEAT-HFpEF trial demonstrated reduced activity levels and no improvement in exercise capacity with isosorbide mononitrate compared with placebo.^36^ Also, beta-blockers should be used cautiously in the absence of a clear indication (eg AF or CAD), as they may exacerbate chronotropic incompetence, which is common in HFpEF and contributes to reduced exercise tolerance.^37^

Regular medication review is important, particularly in frail patients, to minimise polypharmacy and treatment-related adverse effects, such as hypotension and electrolyte disturbance.

### Non-pharmacological management

Exercise intolerance in HFpEF is partly driven by peripheral and skeletal muscle abnormalities, providing a rationale for training interventions. Exercise training can improve quality of life and should be encouraged.^38^, ^39^ The REACH-HFpEF trial is currently evaluating the role of a structured cardiac rehabilitation programme in this population.^40^ Patients should be supported with education, self-management strategies and access to community resources and multidisciplinary HF care. Early assessment of frailty is recommended to guide treatment intensity and inform goals of care.

## For selected patients

### Obesity-targeted therapy (obese-HFpEF phenotype)

Anti-obesity therapy is not yet guideline-directed in HFpEF but is increasingly supported by RCT evidence in patients in whom obesity is likely a driver of symptoms. Weight loss through lifestyle interventions can improve exercise capacity in the short term,^41^ although sustained benefit is often difficult to achieve.

The STEP-HFpEF programme demonstrated that treatment with semaglutide 2.4 mg once weekly, improved symptoms, physical limitations and exercise capacity, in patients with obesity-related HFpEF.^42^, ^43^ Subsequently, the SUMMIT trial evaluating tirzepatide (a dual GLP-1/GIP receptor agonist) reported improvements in health status and fewer HF events, although overall event rates were low, limiting interpretation of clinical outcome effects.^44^ Long-term effects on hard cardiovascular outcomes remain to be established.

In practice, these agents may be considered in patients with HFpEF and obesity with significant functional limitation, with caution in those with frailty or sarcopenia. Monitoring should focus on tolerability (particularly gastrointestinal effects) and appropriate adjustment of concomitant diabetes therapies.

### Management of other comorbidities

Optimisation of comorbidities is fundamental, as most patients with HFpEF have multiple coexisting conditions that drive symptoms and outcomes. AF is highly prevalent in HFpEF and is associated with more advanced symptoms, reduced exercise capacity and increased mortality compared with patients in sinus rhythm.^45^, ^46^ Anticoagulation for thromboembolic prevention should be prescribed according to guideline recommendations, and rate control may improve symptoms. Rhythm control strategies, including catheter ablation, are under investigation. Hypertension, diabetes, CKD, CAD and chronic lung disease should be managed according to their respective guidelines. Screening for sleep-disordered breathing should be considered where clinically appropriate. Overall comorbidity management should be individualised, balancing potential benefits against frailty and treatment burden.

### Supportive and palliative care

Given the chronic, relapsing and progressive nature of HFpEF, patients should have access to multidisciplinary care, including vaccination, rehabilitation and psychosocial support. Supportive and palliative care should be considered early in patients with advanced symptoms or severe frailty.

## Acute management of HFpEF

Acute management of HFpEF focuses on rapid decongestion, haemodynamic stabilisation, and treatment of precipitating factors. Intravenous loop diuretics should be administered early in patients with congestion and titrated to response, while monitoring renal function, electrolytes and fluid balance. Persistent congestion may require higher doses or combination diuretic therapy. Blood pressure should be controlled. Precipitating factors, such as AF, infection, myocardial ischaemia or uncontrolled hypertension, should also be identified and treated.

Following stabilisation, chronic therapies should be initiated or optimised, including SGLT2 inhibitors where appropriate, with early follow-up to reduce rehospitalisation.

## Future directions

Future HFpEF management is likely to evolve along two trajectories: broad, multisystem therapies and phenotype-directed interventions. The benefits of SGLT2 inhibitors likely reflect their effects across multiple pathophysiological pathways, improving cardio-renal-metabolic function and promoting decongestion. Emerging therapies, including anti-inflammatory and metabolic strategies, will likely need to demonstrate similar cross-organ effects to improve outcomes in this heterogeneous syndrome.^47^

Phenotype-directed approaches are beginning to translate into clinical benefit, particularly in obesity-related HFpEF.^42^, ^43^ Incretin-based and combination metabolic therapies have shown promising results, with ongoing studies expected to further define their role.^44^ Other interventions, including catheter ablation of AF, novel antihypertensive strategies, pulmonary vascular therapies, and device-based approaches to reduce LA pressure or enable haemodynamic monitoring, are under investigation. Careful patient selection remains essential, for example, atrial shunt devices may be harmful in patients with latent pulmonary vascular disease.^48^

For healthcare professionals, the immediate challenge is delivery of care. Earlier recognition, equitable access to HF services, improved patient education, and digitally enabled follow-up will be needed to translate therapeutic advances into real-world benefit. Improving outcomes in HFpEF will not only depend on new therapies, but also on designing care pathways that enable earlier diagnosis, consistent treatment, and prevention of the underlying cardiometabolic substrate.

## Conclusion

HFpEF is an increasingly prevalent cardiovascular syndrome that remains under-recognised. A high index of suspicion and proactive diagnostic evaluation are essential, particularly in patients presenting with unexplained exertional dyspnoea and cardiometabolic multimorbidity. Recent RCTs have demonstrated that HFpEF is treatable. Although current therapies primarily reduce HF hospitalisation rather than mortality, they also provide meaningful improvements in symptoms, functional status, and quality of life, emphasising the importance of early recognition, accurate diagnosis, and timely initiation of evidence-based therapy. Improving outcomes in HFpEF will also depend on integrating evolving evidence into routine care, optimising comorbidities, and delivering coordinated, patient-centred management across healthcare settings.

## CRediT authorship contribution statement

**Adam A. Nabeebaccus:** Writing – review & editing. **Rosita Zakeri:** Writing – review & editing, Writing – original draft, Visualization, Funding acquisition, Conceptualization.

## Funding

RZ is supported by a National Institute for Healthcare Research (NIHR) Advanced Fellowship award (NIHR302961). The views expressed in this publication are those of the authors and not necessarily those of the NIHR, NHS, or the UK Department of Health and Social Care.

## Declaration of Competing Interest

RZ: previous advisory fees from AstraZeneca, Boehringer Ingelheim, Johnson & Johnson, and publication fees from SERB pharmaceuticals outside of the current work. AN: no declarations.
